# MicroRNA-205 functions as a tumor suppressor in human glioblastoma cells by targeting VEGF-A

**DOI:** 10.3892/or.2011.1588

**Published:** 2011-12-12

**Authors:** XIAO YUE, PEIGUO WANG, JUN XU, YUFANG ZHU, GUAN SUN, QI PANG, RONGJIE TAO

**Affiliations:** 1 Department of Neurosurgery, Shandong Cancer Hospital, Shandong Academy of Medical Sciences, Jinan 250117; 2 Department of Radiation Oncology, Tianjin Medical University Cancer Institute and Hospital, Tianjin 300060; 3 Department of Neurosurgery, The First People's Hospital of Yancheng affiliated with Nantong University, Yancheng 224000; 4 Department of Neurosurgery, Provincial Hospital Affiliated to Shandong University, Jinan 250021, P.R. China

**Keywords:** glioma, miRNA-205, VEGF-A

## Abstract

MicroRNAs (miRNAs) are endogenously small non-coding RNAs which are key post-transcriptional regulators of gene expression. Deregulation of miRNAs is common in human tumorigenesis. We report that miRNA-205 is significantly down-regulated in glioma cell lines and tissue specimens. Ectopic expression of miRNA-205 induces apoptosis, cell cycle arrest, impairs cell viability, clonability and invasive properties of glioma cells. We further demonstrate that miRNA-205 can specifically suppress expression of VEGF-A by directly interacting with the putative miRNA-205 binding site at the 3′-UTR. Identification of VEGF-A as a direct target for miRNA-205 may imply that miRNA-205 is a novel target for glioma therapy. Taken together, the present study for the first time provides evidence that miRNA-205 is a glioma-specific tumor suppressor by targeting VEGF-A.

## Introduction

MicroRNAs (miRNAs) are a class of endogenous, small non-protein coding single-stranded RNA molecules, which are key post-transcriptional regulators of gene expression in metazoans and plants. In animals, miRNAs have regulatory effects through binding loosely complimentary sequences within the 3′-untranslated regions (3′UTRs) of their mRNA targets (1,2). *In silico* prediction models suggest that miRNAs may be responsible for the regulation of more than one-third of all human genes. Functional studies indicate that miRNAs are important to several fundamental biological processes, including proliferation, apoptosis, development, and cellular differentiation (3,4).

By negatively regulating their mRNA targets to either degradation or translational repression, miRNAs have the capacity to function as either oncogenes or tumor suppressors (2). Recent findings have suggested that miRNAs not only are important biomarkers, but also might be promising therapeutic targets for various diseases (5–8). Emerging evidence has demonstrated that aberrant expression levels of miRNAs are involved in glioblastoma multiforme (GBM) initiation and progression (9). Notably, miR-21 is almost invariably overexpressed in GBM, resulting in enhanced cell motility, migration and decreased apoptosis (10,11). Alternately, important down-regulated miRNAs have also been identified in glioblastoma, such as miR-128 and miR-7 (12,13). It has been demonstrated that miR-128 targets Bmi-1 and reduces cellular proliferation and self-renewal of glioma stem cells (13).

In this study, the expression of miRNA-205 in glioma cell lines and the tissues specimens from glioma patients with certain grades was studied by real-time PCR analysis. Further investigation revealed that in glioma cell lines, miRNA-205 functioned as a tumor suppressor and overexpression of miRNA-205 reduced cell proliferation, induced G0/G1 phase arrest, decreased cell invasive capacity and increased apoptosis. We further demonstrated that miRNA-205 could specifically suppress expression of VEGF-A by directly interacting with the putative miRNA-205 binding site at the 3′-UTR. Our findings will help to elucidate the functions of miRNAs and their roles in tumorigenesis.

## Materials and methods

### Cell lines and tumor specimens

Human glioma cell lines, H4, U87, LN229 and U251, were purchased from Chinese Academy of Sciences Cell Bank. All glioma cell lines were maintained in a 37°C, 5% CO_2_ incubator in DMEM medium supplemented with 10% fetal bovine serum (Invitrogen, CA, USA) and 1% penicillin-streptomycin (Invitrogen). Cells were routinely passaged at 2–3 day intervals. Tissue samples from human glioma and normal brain tissues were obtained from Shandong Cancer Hospital and Institute (Jinan, China). The histopathologic diagnoses were determined using WHO criteria and evaluated by the hospital's pathologist using both morphologic criteria and immunocytochemistry. Written consent of tissue donation for research purposes was obtained from the patients before tissue collection. Twenty-five samples were used for this research with 5 samples for each group, including primary grade pilocytic astrocytomas (WHO I), grade II astrocytoma (WHO II), grade III anaplastic astrocytomas (WHO III), grade IV Glioblastoma Multiforme (WHO IV) and normal brain tissues derived from the temporal lobes and saddle area of the patients with arachnoid cyst (AC) after surgery.

### RNA isolation and real-time quantitative RT-PCR

Total RNA from the frozen tissue specimens and cultured cells was isolated using the TRIzol kit (Invitrogen) following to the manufacturer's instructions. RNA quantity was determined by UV measurement of OD 260/280 nm using the NanoDrop 2000 instrument (Thermo Scientific, FL, USA). To quantitate the expression level of mature miRNA-205, the isolated RNA was reverse transcribed and amplified using the mirVana™ qRT-PCR miRNA detection kit (Ambion) according to the manufacturer's protocol. PCR reactions were performed using an MJ-real-time PCR (Bio-Rad, Hercules, CA, USA) system with the following conditions: 95°C, 10 min for 1 cycle, then 95°C, 15 sec, 60°C, 1 min for 40 cycles. Signals were detected at the end of each cycle. The U6 small nuclear RNA was amplified as a loading control. The primers for this U6 internal control were purchased from Ambio. Relative quantification was conducted using amplification efficiencies derived from cDNA standard curves and obtained relative gene expression. Data were shown as fold change (2^−ΔΔCt^) and analyzed initially using Opticon Monitor Analysis Software V2.02 software (MJ Research, Waltham, MA, USA). Real-time PCR for VEGF-A was performed using the MJ-real-time PCR System (Bio-Rad) with the QuantiTect SYBR Green PCR mixture (Invitrogen). β-actin was used as control. Amplification conditions were: 95°C, 3 min, 95°C, 30 sec, 60°C, 30 sec, 72°C, 40 sec, for 40 cycles, and 72°C, 8 min for extension.

### Transfection

miRNA-205 mimics and inhibitor and non-targeting control were obtained from Dharmacon. Cells were transfected using Lipofectamine 2000 reagent (Invitrogen) at the time of 70% confluent. Transfection complexes were prepared according to the manufacturer's instructions and added directly to the glioma cells to a final oligonucleotide concentration of 50 nmol/l. Transfection medium was replaced 8-h post-transfection.

### MTT proliferation assay

The capacity for cellular proliferation was measured with a 3-(4,5-dimethylthiazol-2-yl)-2,5-diphenyltetrazolium bromide (MTT) assay. U87 and LN229 cells were plated at 10^4^ cells per well in 96-well plates with six replicate wells for each condition, transfected with oligonucleotides, and assayed 48-h post-transfection. The cells were then incubated with 20 μl of MTT (5 mg/ml) for 4 h at 37°C and 200 μl of DMSO was added to solubilize the crystals for 20 min at room temperature. The optical density was determined with a spectrophotometer [Multiskan MK3 (Thermo)] at a wavelength of 570 nm. Cell growth inhibition rates formula is (AC-AT)/ACx100% (AC, Absorbance value of the blank control group; AT, Absorbance value of the experimental group).

### Anchorage-independent growth assay

After 24 h of transfection, U87 and LN229 cells (5×10^2^) were suspended in 2 ml of 0.3% agarose with DMEM medium containing 12% FBS and plated into six-well plates on top of an existing layer of 0.6% agarose prepared with the same medium. The plates were incubated at 37°C in a 5% CO_2_ incubator. After four weeks, cell colonies were fixed with methanol and stained with 0.1% crystal violet for 10 min. Then, the colonies were captured with Olympus SZX12 and Qcapture Pro software (Olympus). Cell colonies >0.1 mm in diameter were counted under a microscope. Each assay was performed in triplicate on four independent occasions.

### Cell cycle analysis

For cell cycle analysis, 48 h after transfection, the adhered cells were obtained by trypsinization and pooled with the floating cells and centrifuged at 1000 rpm for 5 min and then incubated with RNase at 37°C for 30 min. A total of 10^4^ nuclei were examined by a FACS Calibur flow cytometer and DNA histograms were analyzed by Modifit software (Becton Dickinson, Franklin Lakes, NJ, USA). Experiments were performed in triplicate. Results are presented as percentage of cells in each phase.

### Apoptosis assays

The Annexin V-FITC Apoptosis Detection kit I (Abcam, USA) was used to detect and quantify apoptosis by flow cytometry. In brief, cells were harvested 48 h after transfection and collected by centrifugation for 5 min at 800 × g. Cells were resuspended at a density of 1×10^6^ cells/ml in 1X binding buffer, stained with FITC-labeled Annexin V for 5 min and immediately analyzed by FACScan Flow Cytometer (Becton Dickinson, San Jose, CA, USA). The data obtained were analyzed using CellQuest software.

### Transwell invasion assay

Matrigel invasion assay was performed using a 24-well invasion chamber system (BD Biosciences, Bedford, MA) with polycarbonic membrane (diameter: 6.5 mm, pore size 8 μm). Cells were plated on the top of matrigel-coated invasion chambers in a serum-free DMEM. As a chemo-attractant, DMEM containing 20% of FBS was added to the lower compartment of the chamber. The cells were incubated for 48 h. Invasion of cells to the underside of the Matrigel-coated membrane was detected by staining the cells with Mayer's hematoxylin solution and visualizing the cells under a microscope. After staining, cells were counted under a microscope in four random fields (magnification, ×100) and results were expressed in the form of a bar graph. Assays were done in triplicate for each experiment, and each experiment was repeated three times.

### Western blot analysis

Cells were washed with pre-chilled phosphate-buffered saline (PBS) three times. The cells were then solubilized in 1% Nonidet P-40 lysis buffer (20 mM Tris, pH 8.0, 137 mM NaCl, 1% Nonidet P-40, 10% glycerol, 1 mM CaCl_2_, 1 mM MgCl_2_, 1 mM phenylmethylsulfonyl fluoride, 1 mM sodium fluoride, 1 mM sodium orthovanadate, and a protease inhibitor mixture). Total protein lysates were separated by SDS-PAGE. The separate proteins were transferred to PVDF membranes. The blot was incubated with primary antibody detecting VEGF-A (Santa Cruz; 1:1000 dilution), followed by incubation with HRP-conjugated secondary antibody. The specific protein was detected using a super signal protein detection kit (Pierce). After washing with stripping buffer, the PVDF membrane was reprobed with antibody against GAPDH (Santa Cruz, 1:1000 dilution).

### Synthesis of luciferase reporter constructs

Luciferase reporters were generated based on the firefly luciferase expressing vector pGL3-control (Promega). pGL3-WT-VEGF-A-3′UTR-Luc reporter was created by ligation of PCR products of 3′UTR of VEGF-A into the *Xba*I site of the pGL3 control vector. The primers for PCR amplification are: VEGF-A-3′UTR-Forward: 5′-ATC TCA GCA TGC CTG GTC AGT TAC CTA CTA ATA GCG GGC CTG-3′ and VEGF-A-3′UTR-Reverse: 5′-GCC CTG AGT GCT GAG CGA TCA AGT GTC ATT TGA CGT ATC GCT-3′. pGL3-MUT-VEGF-A-3′UTR-Luc reporter was generated from pGL3-WT-VEGF-A-3′UTR-Luc reporter by deleting the binding site for miRNA-205.

### Luciferase activity assay

Cells were seeded in 24-well plates at 5×10^4^ cells per well the day before transfection. Luciferase reporter (500 ng), 50 pmol (miRNA-205 mimics or NC) and 40 ng of pRL-TK were added in each well. Cells were collected 48 h after transfection and analyzed using the Dual-Luciferase Reporter Assay System (Promega) and Centro LB 960 (Berthold).

### Statistical analysis

SPSS10.0 was used for statistical analysis. One-way analysis of variance (ANOVA) and χ^2^ test was used to analyze the significance between groups. The LSD method of multiple comparisons with parental and control vector groups was used when the probability for ANOVA was statistically significant. Statistical significance was determined at P<0.01.

## Results

### miRNA-205 is down-regulated in glioma cell lines and tissue specimens

**Figure 1:**
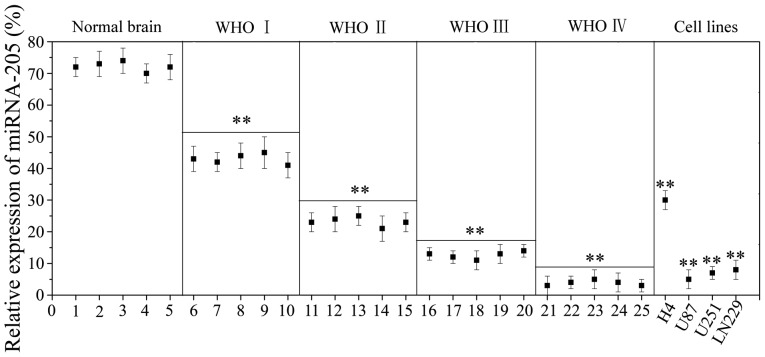
The expression of miRNA-205 in glioma specimens and H4, U87, LN229 and U251 cells. The grade of glioma was evaluated according to WHO criteria as described in Materials and methods. Samples ID 1–5 are from normal brain tissues; ID 6–10 from pilocytic astrocytomas classified to WHO I: ID 11–15 from astrocytoma classified to WHO II; ID 16–20 from anaplastic astrocytomas classified to WHO III; and ID 21–25 from Glioblastoma Multiforme classified to WHO IV. Each sample was divided by a dashed line. Total RNA was isolated from the glioma specimens and glioma cells of H4, U87, LN229 and U251 and real-time PCR was performed to analyze the expression of miRNA-205 as described in Materials and methods. The relative expression of miRNA-205 was expressed as the ratio of the expression level of U6. ^**^P<0.01, as compared to Normal brain tissues group.

Previously, it has been reported that miRNA-205 is down-regulated in breast tumor tissues and breast cancer cell lines (14). However, the expression of miRNA-205 in tissues of glioma patients has not been well documented. To assess its relevance in glioma tumorigenesis, we determined miRNA-205 levels in tumors of different grades compared to normal brain by quantitative RT-PCR (qRT-PCR). The results showed that in normal brain tissues, miRNA-205 exhibited a relative high level expression, whereas the expression of miRNA-205 was significantly (P<0.01) down-regulated in glioma samples (WHO I, II, III and IV). The expression of miRNA-205 was negatively correlated with tumor grade. We also examined expression levels of miRNA-205 in glioma cell lines (H4, U87, LN229 and U251), and normal brain tissues as control. They demonstrated the same expression patterns as miRNA-205 in primary tumors and the normal tissues (Fig. 1). The significant suppression of miRNA-205 expression in tumors and cancer cell lines suggests a tumor suppressor role in glioma.

### miRNA-205 inhibits the proliferation of glioma cells in vitro

The significant reduction of miRNA-205 expression in glioma cell lines and tissue specimens prompted us to explore the possible biological significance of miRNA-205 in tumorigenesis.

**Figure 2:**
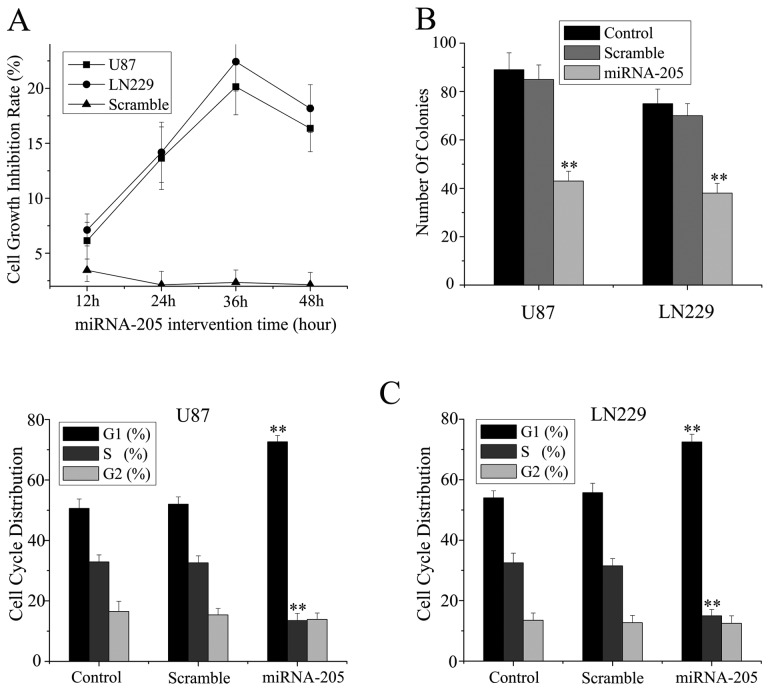
Ectopic expression of miRNA-205 inhibits glioma cell proliferation *in vitro*. (A) The cell proliferation inhibition rates of glioma cell lines of U87 and LN229 after miRNA-205 transfection at certain time points. (B) Anchorage-independent growth assay in U87 and LN229 cells. The colonies were counted and each value represent the mean ± SD from triplicate determinations. Significant differences from the control value are indicated by ^**^P<0.01. (C) U87 and LN229 cells were treated with the miRNA-205 mimics and cell cycle distributions were detected by flow cytometry 48 h later. Percentages of cells in different phases of the cell cycle are shown in the histogram. ^**^P<0.01 vs. control group.

The *in vitro* growth ability of glioma cells was determined by MTT assay. About 13.64±2.85% and 14.18±2.47% inhibition rates of miRNA-205 transfectants in U87 and LN229 cells at 24 h time point were shown in Fig. 2A and the maximum inhibition rate was at 36-h time point. These results imply that miRNA-205 might function as a tumor suppressor in glioma cells *in vitro*.

To determine whether the inhibition of growth induced by miRNA-205 in cells was anchorage-independent, the cells were plated on soft agar 24 h after RNA transfection in U87 and LN229 cells. After four weeks, the cells transfected with miRNA-205 mimics formed significantly fewer colonies on soft agar than control and scramble treated cells (Fig. 2B).

To further examine whether the decrease in proliferation of U87 and LN229 cells reflected a cell cycle arrest, cell cycle progression was analyzed by propidium iodide staining and flow cytometric analysis. The results revealed that U87 and LN229 cells transfected with miRNA-205 mimics had an obvious cell cycle arrest at the G0/G1 phase (Fig. 2C). These results suggest that miRNA-205 induces cell cycle arrest and inhibits proliferation of glioma cells.

### miRNA-205 induces apoptosis in glioma cell lines

**Figure 3:**
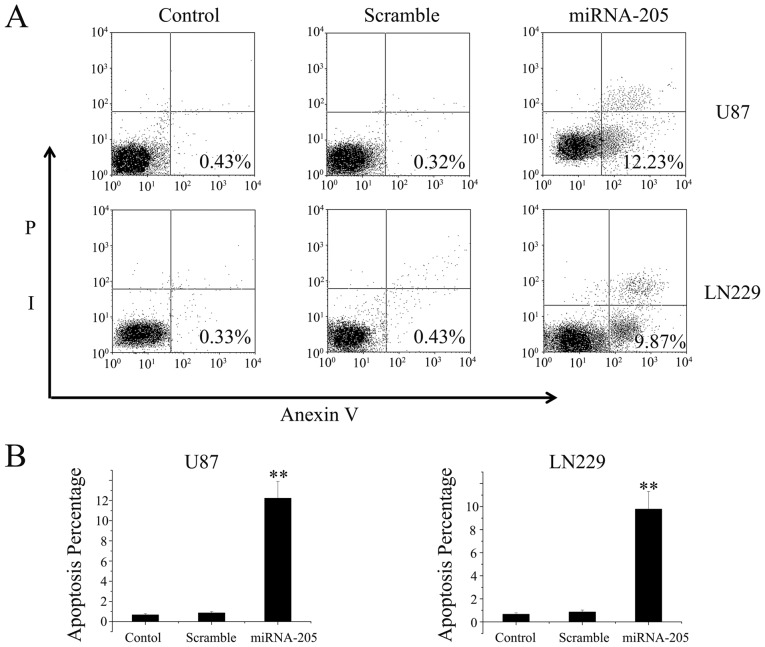
Ectopic expression of miRNA-205 induces apoptosis of U87 and LN229 cells. Flow cytometric analyses of propidium iodide-stained cells were performed in triplicate (A). Percentages of apoptotic cells are shown in the histogram (B). ^**^P<0.01 vs. control group.

We also analyzed the effect of miRNA-205 on apoptosis in glioma cells by conducting Annexin V and PI double staining. The Annexin V-positive early-phase apoptotic cells were significantly increased in cells transfected with miRNA-205 mimics oligonucleotide when compared with untreated or scramble controls cells (Fig. 3A). Percentages of apoptotic cells are shown in the histogram (Fig. 3B). Annexin-V-FITC/PI double staining assay showed that miRNA-205 induced apoptosis of U87 and LN229 cells.

### miRNA-205 depresses the invasion of glioma cells in vitro

**Figure 4:**
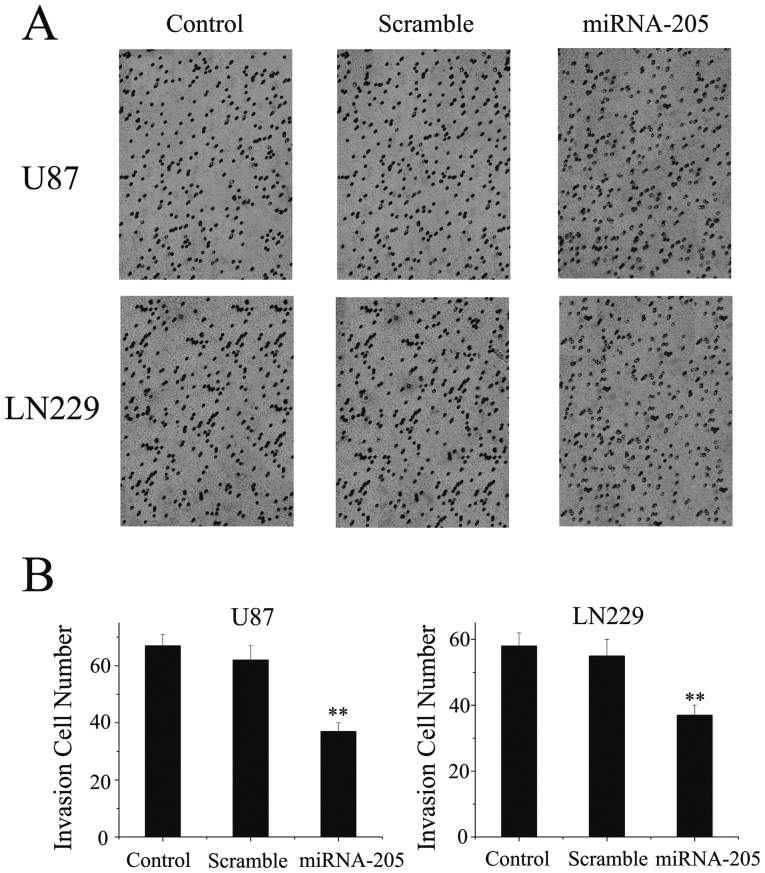
Effects of miRNA-205 on cell invasion ability in glioma cell lines. Cell invasion ability was assessed by a transwell assay after 48 h transfected with miRNA-205 (A). The number of cells that could invade via the membrane is shown (B). ^**^P<0.01 vs. control group.

Invasive growth is an important biological characteristic of malignant glioma cells. To evaluate the impact of miRNA-205 on invasive ability of U87 and LN229 cells, we employed transwell matrigel invasion assay. In U87 cells, miRNA-205 inhibited invasive activity by ~50%, as 36.24±4.12 cells/field invaded the matrigel layer compared to 68.78±3.25 and 64.54±3.47 cells/field in the control and scramble-treated groups, respectively. Similarly, miRNA-205 significantly inhibited invasive activity of LN229 cells, as 36.26±4.02 cells/field invaded the matrigel layer compared to 58.32±3.46 and 56.57±3.12 cells/field in the control and scramble-treated groups, respectively (Fig. 4). These results demonstrate that miRNA-205 significantly reduces glioblastoma cell invasion capacity.

### VEGF-A is a potential target of miRNA-205 in glioma cells

**Figure 5:**
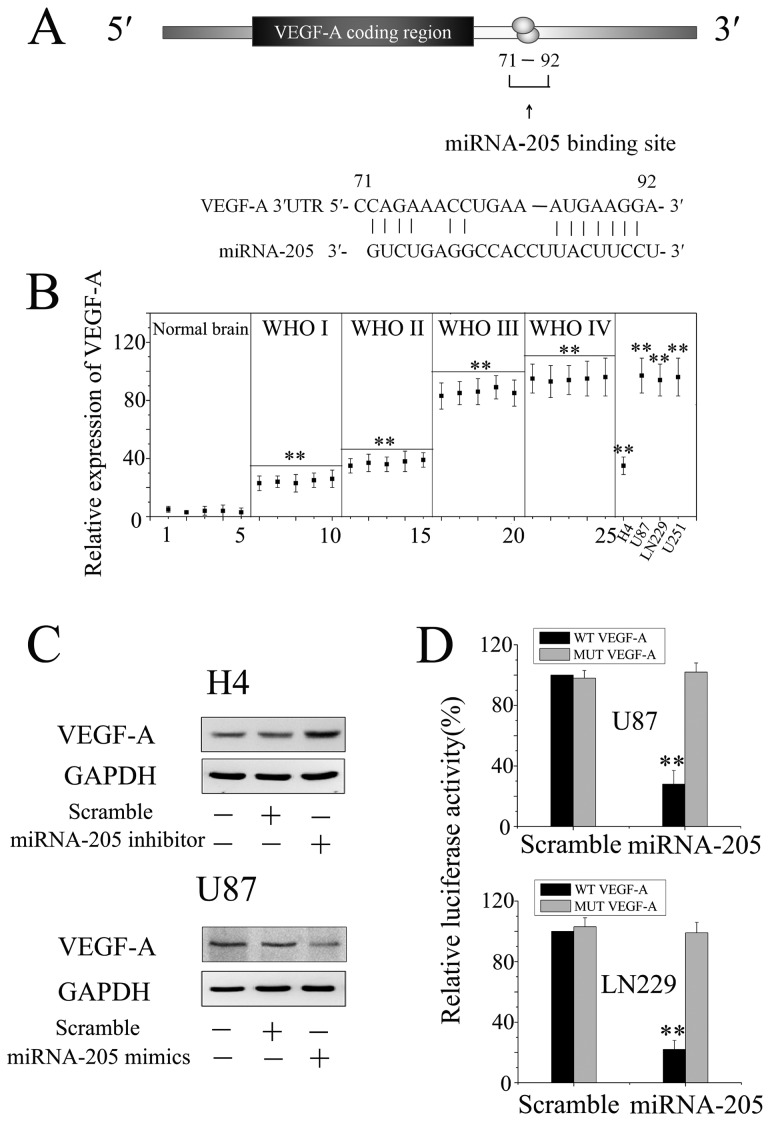
VEGF-A is a direct target gene of miRNA-205. (A) Schematic representation of the putative binding sites in VEGF-A mRNA 3′UTR for miRNA-205. (B) The mRNA expression level of VEGF-A in glioma samples and glioma cell lines. The mRNA expression level of VEGF-A was detected by real-time PCR. The grades of glioma were classified in Materials and methods. The experiments were preformed in triplicate. The relative expression of VEGF-A was the ratio of the expression level of β-actin. ^**^P<0.01, as compared to Normal brain tissues group. (C) Western blot analysis was performed to evaluate the expression level of VEGF-A in H4 and U87 cells, which were transfected with miRNA-205 inhibitor or miRNA-205 mimics, respectively. GAPDH was used as a loading control. (D) pGL3-WT-VEGF-A-3′UTR-Luc and pGL3-MUT-VEGF-A-3′UTR-Luc reporters were transfected into U87 and LN229 cells transfected with miRNA-205 mimics or scramble miRNA. Luciferase activity was determined 48 h after transfection. The activity of Renilla luciferase was normalized to that of the control firefly luciferase in each experiment. The luciferase activity in cells transfected with scramble miRNA was defined as 100%. Error bars represent standard deviation and were obtained from three independent experiments. ^**^P<0.01 vs. scramble miRNA transfected controls.

An obstacle to understanding miRNA function has been the relative lack of experimentally validated targets (15). To understand the molecular mechanisms by which miRNA-205 inhibited glioma cells growth and invasion, we searched for putative miRNA-205 targets as predicted by the commonly cited programs such as TargetScan, miRanda and PicTar and found 3′UTR of VEGF-A containing the highly conserved putative miRNA-205 binding sites (Fig. 5A). We detected the expression of VEGF-A in normal brain, glioma specimens of different grades, and glioma cell lines. Herein, we found that the expression of VEGF-A was significantly elevated with the ascending order of glioma grade (P<0.01), accompanying the decrease of miRNA-205 (Fig. 5B).

Further, we knocked down expression of miRNA-205 in H4 cells, which exhibited elevated level of miR-205, and ectopically expressed miRNA-205 in U87 cells with low endogenous miRNA-205 expression (Fig. 1). Western blot analysis showed that VEGF-A expression was up-regulated in H4 cells with knockdown of miR-205, whereas down-regulated in U87 cells overexpressing miR-205 (Fig. 5C), compared to control or scramble treated cells. Moreover, we created pGL3-WT-VEGF-A-3′UTR and pGL3-MUT-VEGF-A-3′UTR luciferase reporters. Reporter assay revealed that overexpression of miRNA-205 significantly suppressed the activity of pGL3-WT-VEGF-A-3′UTR plasmid in U87 and LN229 cells, without change in luciferase activity of pGL3-MUT-VEGF-A-3′ UTR plasmid (Fig. 5D). These results indicate that miRNA-205 directly modulate VEGF-A expression by binding 3′UTR of VEGF-A in glioma cells.

## Discussion

Glioma is the most common type of malignant primary intracranial tumor. The most frequent and most malignant glioma is glioblastoma [World Health Organization (WHO) grade IV]. Glioblastoma is characterized by the hallmarks of cellular heterogeneity, rapid proliferation, angiogenesis, extensive invasion, hypoxia, necrosis, and infiltration of normal brain tissue (16). Despite recent advances in diagnostics and treatments, the prognosis of patients with glioblastoma has not improved significantly over the past 20 years (17,18). Therefore, there is an urgent need to gain deeper understanding of molecular mechanisms implicated in glioblastoma progression and to develop improved conventional or novel therapeutics.

Knowledge of genetic regulatory mechanisms initiating and maintaining malignancy are essential for understanding malignant cellular transformation, pathologic attributes of cancer, and ultimately, for designing effective strategies for cancer prevention and treatment (19). Genes encoding miRNAs are numerous and an even greater number of predicted miRNAs targets have been identified in the human genome. The breadth of genetic regulatory effects potentially mediated by miRNAs and their central role in diverse cellular and developmental processes has led to the hypothesis that miRNAs might be a novel class of therapeutic targets or an entirely new class of therapeutic agents for the treatment of cancers (20–24). In the present report we detected the miRNA-205 expression level in human glioma samples and found that the decreased expression level of miRNA-205 was negatively correlated with the increased malignancy of glioma. While miR-205 is down-regulated in glioma, breast (25) and esophageal cancer (26), it has been shown to be up-regulated in various types of cancers, including lung cancer, bladder cancer, ovarian cancer and head and neck cancer cell lines (6,27–30). These findings may imply that miRNA-205 could play a dual role in tumorigenicity, depending on tissue type and specific targets. Our further investigation revealed that overexpression of miRNA-205 reduced cell proliferation, induced G0/G1 phase arrest, decreased cell invasive capacity and increased apoptosis in glioma cells. Taken together, the present study for the first time provides evidence that miRNA-205 is a glioma-specific tumor suppressor.

Angiogenesis plays an essential role in tumor growth and progression. A large body of research literature incriminates vascular endothelial growth factor A (VEGF-A) as the most potential mediator of tumor-induced angiogenesis in glioma (31,32). Elevated VEGF-A expression is correlated with both increased tumor microvessel density and increased risk for glioma recurrence and poor prognosis (33,34). In the present study, we showed that the expression of VEGF-A was significantly elevated with the ascending order of glioma grade, accompanying the decrease of miRNA-205. Our study is consistent with other studies that high level expression of VEGF-A plays a critical role in glioma malignancy. It has been reported that knockdown of VEGF-A in cancer cells inhibited cell malignancy and invasion (33). Clinically, accumulating evidence indicates that anti-VEGF-A therapeutic approaches have improved glioma treatment (35). Therefore, identification of VEGF-A as a direct target for miRNA-205 may imply that miRNA-205 is a novel target for glioma therapy.

In the present study, the direct interaction between miRNA-205 and VEGF-A mRNA is supported by several lines of evidence: 1) the 3′UTR of human VEGF-A mRNAs contain a putative binding site for miRNA-205 with significant seed match; 2) miRNA-205 suppresses the activity of a luciferase reporter fused with the 3′UTR of VEGF-A mRNA, 3) miRNA-205 represses the endogenous expression of VEGF-A at both the mRNA and protein level. This finding increases our understanding of VEGF-A regulation in glioma cells.

In conclusion, we showed there is significant low-expression of miRNA-205 in glioma cell lines and tissue specimens. Moreover, we demonstrated that miRNA-205 plays a key role in the malignancy of glioma cells by directly regulating VEGF-A expression. This is the first study demonstrating that miRNA-205 inhibits malignant properties of glioma cells indicating the therapeutic potential of miRNA-205 in the treatment of glioma.
